# Environmental Factors as Key Determinants for Visceral Leishmaniasis in Solid Organ Transplant Recipients, Madrid, Spain

**DOI:** 10.3201/eid2307.151251

**Published:** 2017-07

**Authors:** Nerea Carrasco-Antón, Francisco López-Medrano, Mario Fernández-Ruiz, Eugenia Carrillo, Javier Moreno, Ana García-Reyne, Ana Pérez-Ayala, María Luisa Rodríguez-Ferrero, Carlos Lumbreras, Rafael San-Juan, Jorge Alvar, José María Aguado

**Affiliations:** University Hospital 12 de Octubre of Complutense University of Madrid, Madrid, Spain (N. Carrasco-Antón, F. López-Medrano, M. Fernández-Ruiz, A. García-Reyne, A. Pérez-Ayala, C. Lumbreras, R. San-Juan, J.M. Aguado);; Instituto de Salud Carlos III, Madrid (E. Carrillo, J. Moreno); Hospital General Universitario Gregorio Marañón, Madrid (M.L. Rodriguez-Ferrero);; Drugs for Neglected Diseases Initiative, Geneva, Switzerland (J. Alvar)

**Keywords:** solid organ transplant, solid organ transplantation, visceral leishmaniasis, epidemiology, risk factors, urban outbreak, parasites, Spain, Switzerland, vector-borne infections, *Leishmania infantum*, leishmaniasis

## Abstract

During a visceral leishmaniasis outbreak in an area of Madrid, Spain, the incidence of disease among solid organ transplant recipients was 10.3% (7/68). Being a black person from sub-Saharan Africa, undergoing transplantation during the outbreak, and residing <1,000 m from the epidemic focus were risk factors for posttransplant visceral leishmaniasis.

Visceral leishmaniasis (VL) is an uncommon but potentially fatal complication for solid organ transplant (SOT) recipients (*1**,**2*). Beginning in July 2009, an outbreak of leishmaniasis affected the southwest area of Madrid (*3*). The outbreak was primarily located in Fuenlabrada, which has an annual VL incidence of 21.1 cases/100,000 population (*4*), notably higher than that estimated for the general population in Spain (0.5 cases/100,000 population) (*5*).

Spatial analysis revealed that the highest concentration of cases was in the residential area bordering the park (Bosque Sur) (*6*). A large population of *Lepus granatensis* hares, which serve as a reservoir for *Leishmania infantum*, was present in the area (*7**,**8*), and the *Phlebotomus perniciosus* sand fly in Spain can act as a vector and take blood meals from these hares (*6*,*8*,*9*). Thus, the parkland facilitated the transmission of the leishmaniasis pathogen, which led to the outbreak. This large, urban outbreak provided us the opportunity to analyze the incidence and specific risk factors of VL among SOT recipients.

## The Study

The University Hospital 12 de Octubre in Madrid, Spain, acts as the reference hospital for SOT in South Madrid. We performed a retrospective study of all consecutive adult patients who underwent kidney, liver, or heart transplantation during January 1, 2005–January 1, 2013, and lived in the outbreak area. Patients who underwent SOT before January 1, 2005, were excluded because of the difficulty of ensuing long-term follow-up and the potential of heterogeneity in posttransplant practices. Patients who died or had moved to a different place of residence before outbreak onset were excluded (Technical Appendix Figure 1).

The primary study outcome was the occurrence of VL, the diagnosis of which required confirmation of parasitemia (online Technical Appendix) (*10*). We recorded pretransplant, peritransplant and posttransplant variables and collected various environmental factors prospectively by unblinded, direct interview with the patients. Patients were considered to have frequent contact with dogs if patients reported having dogs at home or taking care of dogs and to have the habit of visiting the park if they reported visiting once a year. The distance between the place of residence and the park was obtained by locating the patient’s home address and measuring the shortest linear distance to the nearest border of the parkland by means of an online mapping tool (Google Maps; Google Inc., Mountain View, CA, USA).

The beginning of the exposure period was set as July 2009 (outbreak onset) for patients who underwent SOT before the outbreak and as the transplant date for those who underwent SOT after outbreak onset. In both cases, the exposure period extended to the date of diagnosis of VL, death, or December 2013. We chose to end the study in December 2013 because the incidence of leishmaniasis decreased thereafter because of the implementation of control measures. The clinical research ethics committee of the University Hospital 12 de Octubre approved the study, and participants provided informed consent.

We analyzed 68 patients (Table 1) for a median follow-up of 4.4 (interquartile range 2.39–6.95) years. VL was diagnosed in 7 patients, yielding a cumulative incidence of 10.3% (95% CI 3.1%–17.5%) and an annual incidence of 2,997 (95% CI 1,213–6,161) cases per 100,000 population. Details on disease pathology and therapy were recorded (Table 2). The mean interval between transplant and diagnosis was 1.34 ± 0.89 years. No patients had visited highly VL-endemic countries.

**Table 1:** Baseline and clinical characteristics of solid organ transplant recipients in study of risk factors for VL, Madrid, Spain, January 1, 2005–January 1, 2013*

Characteristics	Overall cohort, n = 68	VL, n = 7	No VL, n = 61	p value†
Recipient age, y, mean ± SD	51.1 ± 14.2	53.0 ± 15.5	51.0 ± 13.5	0.721
Male sex, no. (%)	48 (70.6)	6 (85.7)	42 (68.9)	0.664
Race, no. (%)				
White	62 (91.2)	5 (71.4)	57 (93.4)	0.112
Black, sub-Saharan African	4 (5.9)	2 (28.6)‡	2 (3.3)	**0.049**
Other	2 (2.9)	0 (0)	2 (3.3)	1.000
Type of SOT, no. (%)				
Kidney	57 (83.8)	6 (85.7)	51 (83.6)	1.000
Liver	8 (11.8)	0 (0)	8 (13.1)	0.587
Heart	3 (4.4)	1 (14.3)	2 (3.3)	0.282
Donor age, y, mean ± SD	46.1 ± 16.2	49.4 ± 17.4	46.3 ± 16.3	0.596
Cold ischemia time, min, median (IQR)	1,005 (630–1,354)	795 (371–1,365)	1,020 (660–1,360)	0.370
No. HLA mismatches, mean ± SD	4.0 ± 1.2	5.0 ± 1.0	4.0 ± 1.2	0.265
DCD donor, no. (%)	18 (26.5)	3 (42.8)	15 (24.6)	0.370
Transplant during the outbreak, no. (%)	41 (60.3)	7 (100.0)	34 (55.7)	**0.037**
Induction therapy, no. (%)				
Basiliximab	22 (32.4)	0 (0)	22 (36.1)	0.087
Antithymocyte globulin	24 (35.3)	4 (57.1)	20 (32.8)	0.233
None	22 (32.4)	3 (42.8)	19 (31.1)	0.673
Maintenance immunosuppression, no. (%)				
Steroids	56 (82.4)	7 (100.0)	49 (80.3)	0.338
Calcineurin inhibitors	60 (88.2)	6 (85.7)	54 (88.5)	1.000
Mycophenolate mofetil/mycophenolic acid	47 (61.8)	4 (57.1)	43 (70.5)	0.668
mTOR inhibitors	10 (14.7)	1 (14.3)	9 (14.8)	1.000
Complications in the first year after SOT, no. (%)				
Acute graft rejection	19 (27.9)	2 (28.6)	17 (27.9)	1.000
CMV infection	21 (30.9)	4 (57.1)	17 (27.9)	0.189
Bacterial infection	60 (88.2)	6 (85.7)	54 (88.5)	0.190
Environmental factors				
Frequent contact with dogs, no. (%)	26 (38.2)	3 (42.8)	23 (37.7)	1.000
Habit of visiting the park, no. (%)§	19 (31.7)	3 (50.0)	16 (29.6)	0.369
Distance from patient’s residence to park, m, median (IQR)	1,220 (849–1,865)	399 (261–985)	1,370 (974–1,880)	**0.001**

*CMV, cytomegalovirus; DCD, donation after circulatory death; HLA, human leukocyte antigen; IQR, interquartile range; mTOR, mammalian target of rapamycin; SOT, solid organ transplant; VL, visceral leishmaniasis. †The p values refer to the comparison between patients with and without visceral leishmaniasis. Significant values are in bold. ‡The country of birth of both patients with posttransplant VL was Equatorial Guinea. §Data available for 60 patients.

**Table 2:** Disease characteristics, demographics, clinical characteristics, therapy, and outcomes of 7 solid organ transplant recipients with VL, Madrid, Spain, January 1, 2005–January 1, 2013*

Characteristics	Patient no.						
	1	2	3	4	5	6	7
Sex	M	M	M	M	M	F	M
Race	Black	Black	White	White	White	White	White
Linear distance from patient’s residence to park, m	794	399	261	1,240	985	233	358
Age at transplant, y	35	34	76	55	68	49	52
Type of SOT	Kidney	Kidney	Kidney	Kidney	Kidney	Heart	Kidney
Donor *Leishmania* spp. serostatus	Negative	NP	Negative	NP	Negative	Negative	Negative
Pretransplant recipient *Leishmania* spp. serostatus	NP	NP	Negative	Positive	Negative	Negative	Negative
Date of transplant	2011 Feb 11	2010 Jan 22	2010 Mar 10	2010 Jul 7	2009 Dec 29	2010 Sep 5	2010 Apr 15
Interval from transplant to VL diagnosis, y	1.17	2.44	0.25	1.4	0.17	1.81	2.21
Fever at admission	Yes	Yes	No	Yes	Yes	Yes	Yes
Pancytopenia	Yes	Yes	Yes	Yes	Yes	Yes	Yes
Splenomegaly	Yes	Yes	Yes	Yes	Yes	Yes	Yes
Serologic testing results for *Leishmania* spp.	Positive	Negative	Negative	Positive	Negative	Positive	Negative
Presence of amastigote forms in bone marrow sample	Positive	Positive	Positive	Positive	Positive	Positive	Positive
PCR assay results of bone marrow sample	NP	NP	NP	Negative	NP	NP	NP
NNN culture results of bone marrow sample	Positive	NP	Positive	Negative	Negative	Positive	Negative
Initial therapy	L-AmB	L-AmB	L-AmB	L-AmB	L-AmB	L-AmB	L-AmB
Relapse	Yes	No	Yes	Yes	No	No	No
Outcome	Renal failure	Graft loss	Cured	Graft loss	Cured	Cured	Cured

*L-AmB, liposomal amphotericin B; NNN, Novy-McNeal-Nicolle; NP, not performed; SOT, solid organ transplant; VL, visceral leishmaniasis.

**Figure 1:**
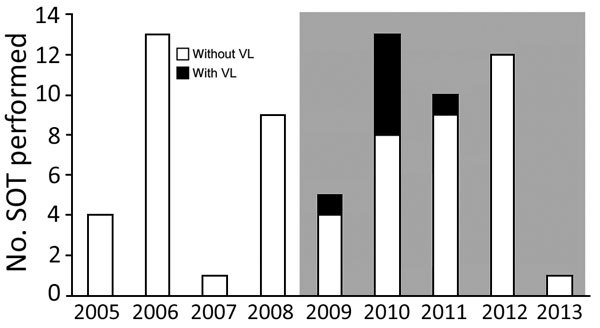
Distribution of VL among solid organ transplant recipients, Madrid, Spain, January 1, 2005–January 1, 2013. Columns represent the number of solid organ transplant procedures performed each year at the University Hospital 12 de Octubre among patients permanently residing in Fuenlabrada, the nearby city affected by the outbreak. Gray shading indicates outbreak period. SOT, solid organ transplant; VL, visceral leishmaniasis.

Black sub-Saharan African SOT recipients were more likely than other recipients to become affected by VL (relative risk 6.40, 95% CI 1.76–23.29, p = 0.049) (Table 1). All 7 episodes of VL occurred in patients who underwent transplantation during the outbreak period (Figure 1).

**Figure 2:**
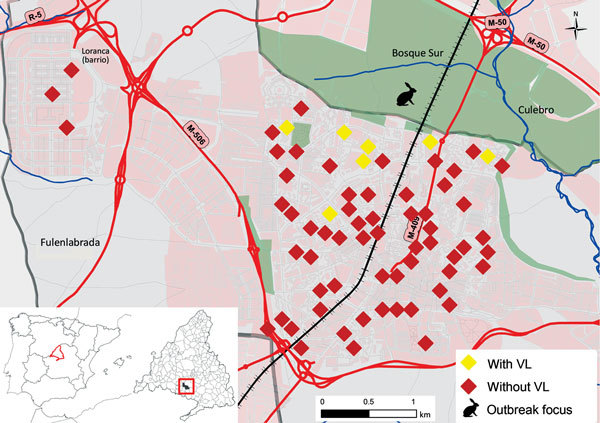
Spatial distribution of solid organ transplant recipients in the southwest area of Madrid, Spain, in relation to park that was focus of visceral leishmaniasis (VL) outbreak, January 1, 2005–January 1, 2013. Map inset shows the location of the outbreak in relation to the rest of Spain. VL, visceral leishmaniasis.

The median distance between the place of residence and the park was significantly shorter for recipients with VL (399 m) than for those without (1,370 m; p = 0.001) (Figure 2; Technical Appendix Figure 2). We explored the predictive accuracy of this variable by establishing the optimal cutoff value with the area under the receiving operating characteristic curve analysis. Recipients living <1,000 m from the park (26.1%, 6/23) had a higher incidence of VL than recipients living >1,000 m away (2.2%, 1/45; relative risk, 11.74, 95% CI 1.50–91.78; p = 0.005). At 4 years, a lower percentage of the SOT recipients living <1,000 m from the park were free from VL than those living >1,000 m away (61.0% vs 98%; p = 0.001 by log-rank test) (Technical Appendix Figure 3).

Our study suggests that the incidence of VL in SOT recipients is notably higher than that in the general population (*11*). Acquisition of the parasite most likely occurred posttransplant because all but 1 recipient affected with VL (from whom serum samples could be recovered) were seronegative for *Leishmania* spp. before transplantation.

Our findings suggest that environmental factors might be crucial in modulating the incidence of VL in immunocompromised hosts, such as SOT recipients; the distance from the patient’s residence to the focus of the outbreak (*6**,**7*) emerged as a key risk factor. The median distance between the park and the homes of recipients with posttransplant VL was <500 m; the maximum flight distance of female sand flies is 600 m (*12**,**13*). Therefore, persons living within this radius had a higher chance of being bitten by the VL vector. A similar association was described for the general population during this outbreak (*6*).

Undergoing transplantation during the outbreak period was another risk factor for VL. This finding suggests that, in the case of an outbreak in a country with low baseline incidence, pretransplant screening of patients listed for SOT for VL-specific antibodies should be considered and repeated during the posttransplant period for the prompt detection of de novo infection. Recipients should also receive specific counseling to reduce the risk of being bitten by sand flies. In addition, treating physicians must maintain a low threshold of suspicion for VL for persons on immunosuppressive therapy during a VL outbreak.

We found that 28% of posttransplant VL cases occurred in black recipients born in sub-Saharan Africa, even though this subgroup only represented 2.4% of the overall population in the affected area (*14*). An association between sub-Saharan African ethnicity and VL has also been reported in the general population (*4*). No apparent relationship was found between the race of the patient and the frequency of parkland visits. Both black recipients in question came from Equatorial Guinea, a country not considered endemic for leishmaniasis by the World Health Organization (*15*). Therefore, the potential association between genetic susceptibility and posttransplant VL warrants further investigation.

Limitations of this study include the small sample size and that interviewers were not blinded to the diagnosis of VL. However, the objective nature of the questionnaire minimized the potential risk for bias. When assessing degree of exposure to sand flies, we used only indirect variables (i.e., distance between the patient’s residence and park, habit of visiting the park) as surrogate measures. Regarding the distance from the park, only linear distances were assessed without considering the potential presence of physical obstacles in the sand fly flight trajectory. Because of these limitations, our findings must be interpreted with caution.

## Conclusions

Our study indicates several risk factors (being black and from sub-Saharan Africa, having an SOT during the outbreak, and living <1,000 m from the outbreak focus) useful for helping physicians treat SOT recipients during a VL outbreak. Doctors should select the patients with these risk factors for counseling to minimize their exposure to vectors and active monitoring for prompt diagnosis.

Technical Appendix Methods used to confirm visceral leishmaniasis (VL) diagnosis, flow chart of solid organ transplant (SOT) recipients included in the study, box-whisker plot comparing linear distances from outbreak focus area (park) to residences of the SOT recipients with and without VL, and Kaplan-Meier curve analysis showing differences in VL disease onset in SOT recipients on the basis of distance from park.
