# Supplementary material for: Environmental Factors as Key Determinants for Visceral Leishmaniasis in Solid Organ Transplant Recipients, Madrid, Spain
Source: Emerg Infect Dis. 2017 Jul;23(7):1155–9. doi: 10.3201/eid2307.151251 (PMC5512489; doi:10.3201/eid2307.151251)
Supplement: Technical Appendix — Methods used to confirm visceral leishmaniasis (VL) diagnosis, flow chart of solid organ transplant (SOT) recipients included in the study, box-whisker plot comparing linear distances from outbreak focus area (park) to residences of the SOT recipients with and without VL, and Kaplan-Meier curve analysis showing differences in VL disease onset in SOT recipients on the basis of distance from park. [file 15-1251-Techapp-s1.pdf]

Article DOI: https://dx.doi.org/10.3201/eid2307.151251

# Environmental Factors as Key Determinants for Visceral Leishmaniasis in Solid Organ Transplant Recipients, Madrid, Spain

## Technical Appendix

### Microbiological Procedures for Parasitological Confirmation

Serology was performed by both a commercial microwell ELISA test (SciMedx Corporation, Denville, NJ, USA) and an rK39 antigen-based immunochromatographic test (InBios International Inc., Seattle, WA, USA) following the manufacturer's instructions. Parasitological diagnosis was based on histologic or Giemsa-stained smear examination for the presence of amastigote forms in bone marrow samples, on culture with Novy-McNeal-Nicolle medium (Francisco Soria Melguizo, Madrid, Spain) to detect promastigotes, or on the DNA detection by means of an in-house PCR assay that amplifies a 145-bp fragment from *Leishmania infantum* kinetoplast DNA. Retrospective serologic testing for *Leishmania* spp. was performed with stored serum samples collected before transplantation from those recipients who developed visceral leishmaniasis and their corresponding donors.

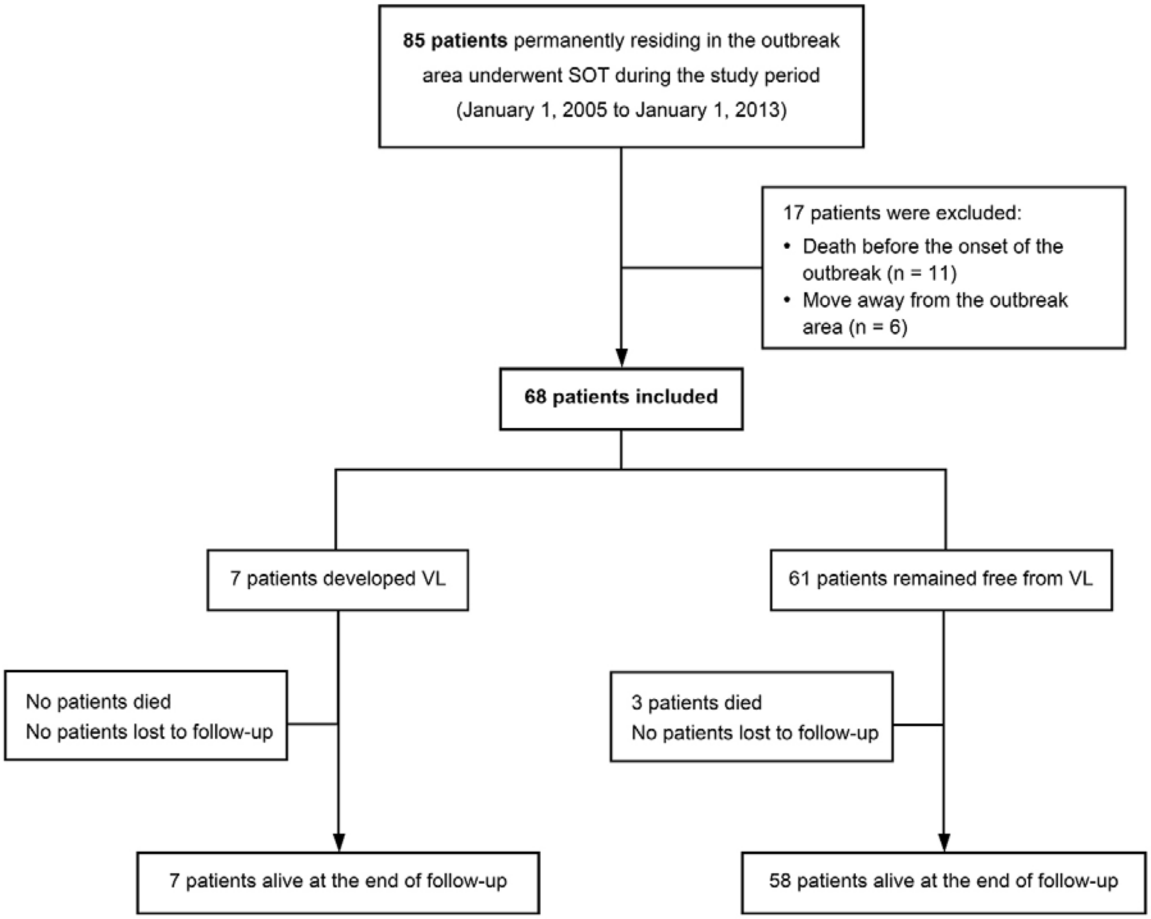


**Technical Appendix Figure 1.** Patient flow diagram. SOT, solid organ transplantation; VL, visceral leishmaniasis.

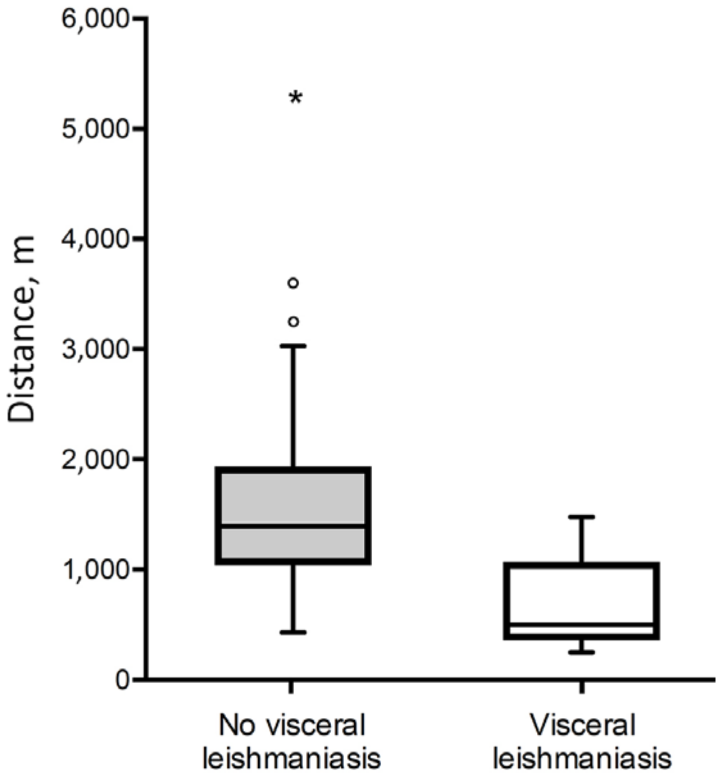


**Technical Appendix Figure 2.** Linear distance between solid organ transplant recipient's place of residence and nearest border of the park. Distance (m) is depicted with a box-whisker plot that shows the median, interquartile range (box), percentiles 5 and 95 (whiskers), and outlier/extreme values (dots). * p = 0.001.

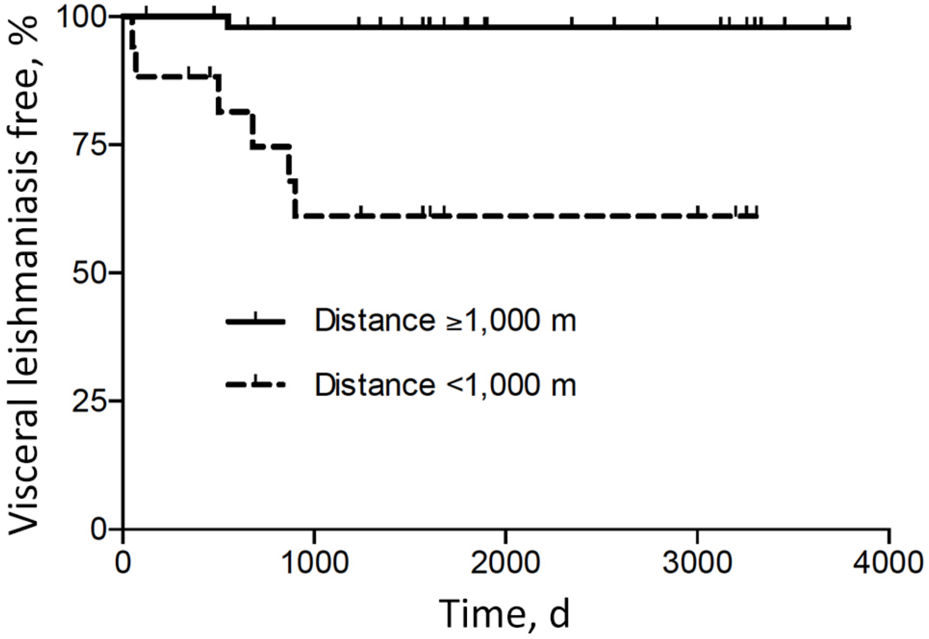


**Technical Appendix Figure 3.** Kaplan-Meier curves depicting the percentage of solid organ transplant recipients free from visceral leishmaniasis by their linear distance from the parkland over the course of the study. The parkland was the area identified as the epidemic focus of the outbreak. In the event of a death in a person without visceral leishmaniasis, the data was censored at the date of death. At 4 years, a reduced percentage of the solid organ transplant recipients living <1,000 m away from the parkland were visceral leishmaniasis free (log-rank test p = 0.001).
